# Predicting Outcomes of Rat Vascularized Composite Allotransplants through Quantitative Measurement of Chimerism with PCR-Amplified Short Tandem Repeat

**DOI:** 10.1155/2020/9243531

**Published:** 2020-02-04

**Authors:** Hui-Yun Cheng, Xiao-Ting Huang, Chih-Fan Lin, Nidal F. AL Deek, Ling-Yi Shih, Cheng-Hung Lin, Fu-Chan Wei

**Affiliations:** ^1^Center for Vascularized Composite Allotransplantation, Linkou Chang Gung Memorial Hospital, Kueishan Taoyuan 333, Taiwan; ^2^Department of Plastic and Reconstructive Surgery, Linkou Chang Gung Memorial Hospital, Kueishan Taoyuan 333, Taiwan; ^3^College of Medicine, Chang Gung University, Kueishan Taoyuan 333, Taiwan

## Abstract

Chimerism has been associated with the induction and maintenance of tolerance to vascularized composite allotransplants (VCA). Although most VCA studies have examined chimerism using flow cytometry, we proposed that precision in the measurement of chimerism may be better approximated when complimentary polymerase chain reaction (PCR) is applied to a specific short tandem repeat (STR). We identified a STR, D10Rat25, which exhibited a ~20 bp difference in length between two rat strains (BN and LEW) often utilized as the donor and recipient in many allotransplantation studies. D10Rat25 was PCR-amplified and quantified with capillary electrophoresis. With pure LEW and BN DNA, a standard curve was constructed to measure chimerism with good linearity. When applied to rat VCA, the relationship between systematic (in peripheral blood) or local (at specific organ/tissues) chimerism to allograft outcomes was noted. We found that peripheral chimerism was elevated by up to ~9% postoperative month 1 (POM 1) but then reduced regardless of the final VCA outcome. However, differences in VCA skin chimerism between early rejection and POM 1 (shown as *Δ*Chimerism_POM1-ER_) were notable with respect to VCA outcomes. ROC analysis identified the optimum cutoff value as 17.7%. In summary, we have developed a reliable method to quantify the percentage of BN cells/DNA in BN-LEW chimeras. The detection limit was characterized, and the acquired data were comparable with flow cytometry. This method can be applied to solid organ and composite tissue allotransplantation studies.

## 1. Introduction

Allotransplantation of solid organs (SOT), hematopoietic stem cells (HSCT), and vascularized composite tissues (VCA) has shown promise in treatment of organ failure, as well as restoration of form and function. However, the lifelong administration of immunosuppressants to suppress rejection results in undesired side effects and consequently prevents wider application of allotransplantation. Therefore, achieving “transplantation tolerance” in the recipients to ensure that allografts function well without immunosuppressants has been an active pursuit in the transplantation field.

Although many VCA studies have shown that development of chimerism, defined as the coexistence of cell populations derived from the donor and recipient, is associated with induction of tolerance to allotransplants [1, 2], conflicting results were reported. For example, Siemionow et al. showed that recipients who developed tolerance to hindlimb VCA had 30% multilineage chimerism in peripheral blood at postoperation day (POD) 120 [3] and up to 15% [4] chimerism when measured at POD 400. However, Rahhal et al. demonstrated peripheral chimerism lasted transiently for about 6 weeks in tolerant VCA recipients [5]. Furthermore, Foster et al. reported that chimerism level correlated with occurrence of rejection; that is, <20% and >60% donor chimerism led to rejection and acceptance of the hindlimb allografts, respectively [6], although evidence suggesting dissociation between chimerism and VCA outcome was also presented [7]. We suspect such conflicts may stem from technical variations borne to flow cytometry, the technology commonly applied to measure chimerism in peripheral blood in VCA studies. Flow cytometry depends on the antibody against a specific donor molecule, so the results may have been confounded by unspecific antibody binding and cell autofluorescence. Moreover, it is subjected to potential intersite and operator variability even with standardized staining and gating protocols, especially when the target cell population is in low abundance [8]. Therefore, we hypothesized precision in measurement can be improved when another method is included.

The polymerase chain reaction (PCR) is a sensitive and specific technique for detecting small molecular changes. PCR amplification of short tandem repeats (STR), which is already used to assess clinical chimerism following HSCT [9], could be useful for VCA studies as well. STR (also called microsatellites) are repetitive sequences of two to seven base pairs in genomic DNA. They have high degrees of heterozygosity and polymorphism and are shown to be good indicators of individuality [10]. Herein, we demonstrate that a PCR-based method would help to achieve precise measuring chimerism of a common rat allotransplantation combination, i.e., Brown Norway (BN) donor to Lewis (LEW) recipient.

## 2. Materials and Methods

### 2.1. Animal

Male 8-12-week-old BN (RT1n) and LEW (RT1l) rats were purchased from the National Laboratory Animal Center, Taiwan. They were housed in pyrogen-free conditions under controlled temperature and lighting cycles with water and commercial rat chow freely available at the Chang Gung Memorial Hospital Animal Center. All experiments were conducted in accordance with the Guide for the Care and Use of Laboratory Animals of the National Institutes of Health and following the Institutional Animal Care and Use Committee (IACUC) protocol authorized by Chang Gung Memorial Hospital, Taiwan.

### 2.2. Genomic DNA Isolation

At designated time points, 0.25 ml blood was drawn from the rat tail and subjected to erythrocyte lysis by ACK (ammonium-chloride-potassium) solution. Genomic DNA isolation from the white blood cells was performed with commercial QIAamp DNA Blood Mini Kit (QIAGEN, Valencia, CA) following the manufacturer's instructions. Collected tissues were saved in -80°C and grinded with liquid nitrogen followed by DNA extraction with QIAamp DNA Mini Kit (QIAGEN, Valencia, CA). Concentration of the collected DNA was measured by OD_260_. Only the DNA with the ratio of OD_260_/OD_280_ higher than 1.8 was included to ensure the purity. The integrity of genomic DNA was inspected by 0.8% agarose gel electrophoresis.

### 2.3. STR-PCR and QIAxcel Analysis

PCR amplification was performed in either 2720 Thermal Cycler or GeneAmp® PCR System 9700 (Applied Biosystems, Foster City, CA), with HotStarTaq Plus Master Mix Kit (QIAGEN, Valencia, CA). One hundred nanogram of template DNA was subjected to 30 cycles of PCR amplification with denaturation at 95°C for 30 s, annealing at 60°C for 40 s, and extension at 72°C for 20 s in the total volume of 40 *μ*l and the presence of 20 pmol primers. Primer sequences were listed as follows: D10Rat25: forward: 5′-CAGGGCACATGAGACAGTTG-3′, reverse: 3′-AAATGGGCTGGAGTAACACG-5′; RT1B*β* [11]: forward 5′-CGCAGGGGATTTCGTATT-3′, reverse 3′-TCTGCCTCCAGGGGTGG-5′. Each PCR product was resolved by either 12% polyacrylamide gel electrophoresis or capillary electrophoresis with QIAxcel DNA High Resolution Kit on QIAxcel Advanced System (QIAGEN, Valencia, CA).

### 2.4. Sequencing of STR-PCR Product

Sanger sequencing reactions were performed on amplified PCR products with BigDye® Terminator v3.1 Cycle Sequencing Kit (Thermo Fisher, Waltham, MA, USA) in the presence of STR-specific primers following the manufacturer's instructions. The reaction mixture was purified by ethanol/EDTA precipitation, followed by sequence analysis on the ABI PRISM® 96-capillary 3730xl DNA Analyzer (Thermo Fisher, Waltham, MA, USA).

### 2.5. PCR Product Quantitation

A series of templates composed of different ratios of BN and LEW DNA, including 0%, 0.1%, 0.2%, 0.4%, 0.8%, 1%, 2%, 4%, 8%, 10%, 20%, 40%, 60%, 80%, and 100% BN DNA, were prepared as templates for D10Rat25-PCR. Following PCR amplification, capillary electrophoresis was performed to resolve the products, whose sizes were determined by referencing the signals of a QX Alignment Marker and QX DNA Size Marker (QIAGEN, Valencia, CA). For each template, signal intensities stemming from BN and LEW D10Rat25 were obtained, and the ratio of BN/(BN+LEW) was derived. The real percentage of DNA was plotted against the corresponding signal ratio, and a standard curve was derived by linear regression with a correlation coefficient (*R*^2^).

### 2.6. Flow Cytometry

White blood cells from venous blood were collected following erythrocyte lysis and stained with the PE-conjugated anti-RT1Ac (Bio-Rad, Hercules, CA, USA). Antibody-bound cells were analyzed by FACSCanto II Flow Cytometer (BD Biosciences, San Jose, CA).

### 2.7. Bland-Altman Plot

It was applied to find the degree of agreement between flow cytometry and D10Rat25-PCR [12]. Leukocytes were isolated from BN and LEW rat blood and mixed with a series of 0%, 5%, 10%, 25%, 50%, and 100% BN cells. The same preparation of cell mixtures was then divided and subjected to either flow cytometry to examine BN-specific RT1Ac expression or D10Rat25-PCR following DNA isolation. Differences between the two measurements were then plotted on the *y*-axis against their means on the *x*-axis. Values of the 95% limits of agreement were derived from (mean) ± 1.96^∗^(SD) of the reading differences.

### 2.8. Rat VCA Model

#### 2.8.1. Donor Operation

A circle incision was performed in the inguinal region; the skin flap with 3 cm in dimension was elevated, based on superficial epigastric vessels. The femoral vessels were identified and prepared. The deep muscular branch of the femoral vessels was preserved to maintain circulation to the femur. The gluteus maximus, tensor fascia latae, and biceps femoris muscles were incised; thigh muscles were sectioned medially along the gracilis muscle from the knee to the groin. The vastus muscles (medialis, intermedius, and lateralis) and rectus femoris muscles were included in the graft and separated from gluteus muscles at the joint capsule. The femoral head was dissected out of the acetabulum, and the femur was fully preserved. The graft was flushed with heparinized saline and was separated from the donor when the recipient site was prepared.

#### 2.8.2. Recipient Operation

The inguinal area defect was created matching the donor's skin flap size. The superficial epigastric vessels were ligated, and the graft circulation restored by 10/0 nylon microanastomoses between the recipient and donor femoral vessels. The graft tissues were inset in the lower abdominal area and then covered and closed with the donor skin flap.

### 2.9. Follow-Up

VCA were evaluated daily and recorded with photography. According to the progression, rejection was defined as “early” when mild erythematous change was observed for two days in a row, whereas exudation, blister formation, and hair loss on ≥50% of VCA area signified “late rejection.” The rejection day was defined when 80% of skin area showed dark purplish discoloration with blister formation and major hair loss.

### 2.10. Skin Grafting

Dorsal cutaneous defects, superficial to the panniculus carnosus, were created in recipients with long-term surviving VCA for insetting BN- or Sprague-Dawley- (SD-) origin full-thickness, tail skin grafts (2 cm × 1 cm). These were fixed with tie-overs for 5 days and were then evaluated daily for another 30 days. Rejection was suggested if erythema, edema, scaling of the skin, hair loss, epidermolysis, and desquamation occurred. Destruction of more than 80% of the graft defined complete rejection.

### 2.11. Statistical Analysis

Statistical analysis was performed with SAS 9.4 and IBM SPSS Statistics. Groups were compared with nonparametric Mann-Whitney *U* analysis. Data were presented with either averages and standard deviations or box-and-whisker plots. The asterisk sign designates the statistically significant difference (*p* < 0.05).

## 3. Results

### 3.1. PCR-Amplified Signal of D10Rat25 Reflected the Template Composition

**Figure 1:**
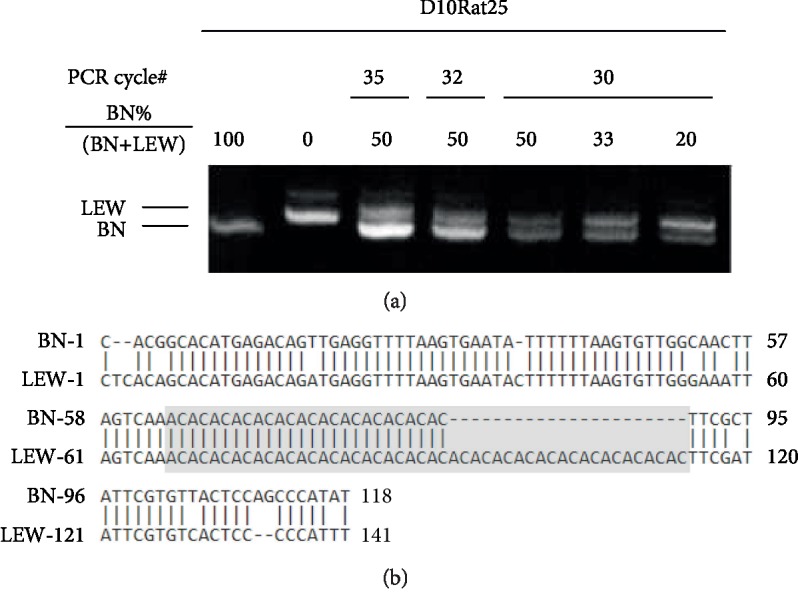
(a) D10Rat25 from BN and LEW rats can be resolved by traditional electrophoresis following PCR amplification. Thirty cycles of amplification reflected template composition [BN%/(BN+LEW)] most faithfully. (b) Sequences of the PCR products confirmed their STR characteristics. The gray area marks the short tandem region.

The online Rat Genome Database (http://rgd.mcw.edu/) provided detailed information on available rat STR [13]. As shown in Table 1, four STR with size differences between the BN and LEW strains were chosen for further characterization. They were tested by PCR amplification of a mixture of equal amount of genomic DNA isolated from naïve BN/SsNNarl and LEW/SsNNarl rats, respectively. Among these STR, amplified D10Rat25 can be fully resolved by conventional gel electrophoresis and roughly reflected the template composition (Figure 1(a)). The product was then sequenced and confirmed to be the targeted STR (Figure 1(b)). Testing with different amplification cycles (30, 32, and 35) on the same BN and LEW DNA mixture showed the products from the 30-cycle PCR reflected the template composition most faithfully. All subsequent experiments were then conducted with 30 amplification cycles.

**Figure 2:**
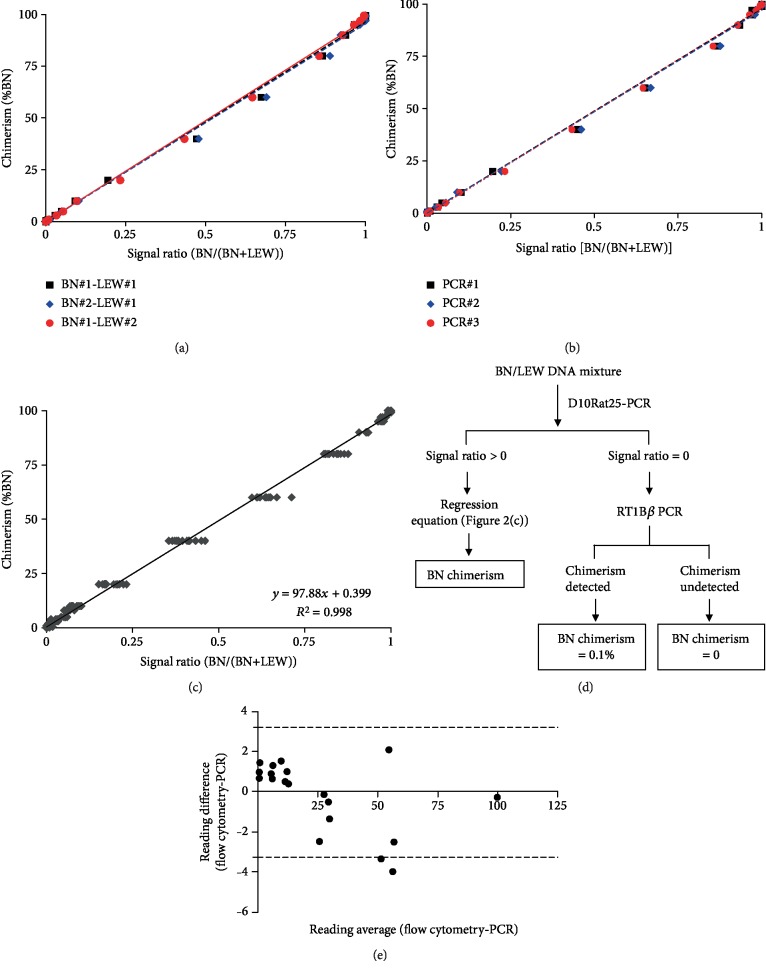
(a) DNA templates derived from 2 BN and 2 LEW rats yielded good linearity between %BN chimerism and derived signal ratio [BN/(BN+LEW)] of amplified D10Rat25. (b) One set of DNA template amplified with three PCR systems showed good consistency. (c) Standard curve derived from 14 datasets from different rats and PCR systems. (d) Workflow to acquire BN chimerism in unknown samples. (e) Good data agreement between flow cytometry and D10Rat25-PCR. Four sets of cell mixtures were subjected to chimerism analysis by flow cytometry with RT1Ac antibody and STR-PCR with D10Rat25. The dotted lines represent 95% limits of agreement (please see text).

At 3~5 bp resolution of the QIAxcel Advanced System, sizes of D10Rat25 in BN and LEW appeared at approximately 130 bp and 158 bp, respectively. The percentage of BN DNA in the template exhibited a strong correlation with the amplified D10Rat25 signal ratio ([BN/(BN+LEW)]) (*R*^2^ > 0.99). Data points from the three batches of the DNA series superimposed on one another, suggesting high reproducibility (Figure 2(a)). Additionally, data were highly consistent among three different PCR systems applied to a single set of DNA templates (Figure 2(b)). These data demonstrated that D10Rat25 can be used to accurately monitor the percentage of BN DNA in BN-LEW chimeras. It would also be feasible to apply this method across different research groups that utilize different PCR systems for data comparison purposes. We then pooled together 14 sets of data that were collected from different animals, performing at different time points by two PCR systems to produce a standard curve (Figure 2(c)).

### 3.2. Detection Limit

As low as 0.4% BN DNA can be reliably detected by D10Rat25-PCR with different batches of DNA mixture and PCR systems. For lower degrees of chimerism, we incorporated a previously reported BN-specific MHC class I molecule, RT1B*β* [11], and found that amplification of RT1B*β* in the template for 60 cycles reliably detected the presence of 0.1% BN DNA. However, quantitative analysis of PCR-amplified RT1B*β* by capillary electrophoresis revealed a moderate correlation between BN DNA percentage and signal intensity (*R*^2^ = 0.6). Thus, RT1B*β* provides a robust qualitative indicator of low chimerism. We then designed a standard workflow to derive the chimerism level from genomic DNA of blood and tissue samples derived from LEW rats with BN allotransplants. As shown in Figure 2(d), genomic DNA was first subjected to D10Rat25-PCR. If the signal ratio derived from capillary electrophoresis of the QIAxcel system was higher than 0, the ratio fit the standard curve shown in Figure 2(c), and the BN chimerism of the sample was derived. However, if the signal ratio was 0, the DNA was subjected to RT1B*β* PCR for 60 cycles followed by capillary electrophoresis. A positive amplified signal suggests that low degrees of BN chimerism (microchimerism) existed in the sample.

### 3.3. Agreement between Flow Cytometry and D10Rat25-PCR Methods

The aforementioned data were acquired using templates composed of genomic DNA from BN and LEW rats with known ratios. Since chimerism is generally defined by the cell ratio and measured by flow cytometry, it is worth evaluating whether the current PCR methodology can be applied to cell mixtures. It is also critical to examine whether measurements made by flow cytometry and STR-PCR are consistent with each other. To that end, we prepared mixtures of LEW and BN cells with known ratios and analyzed chimerism with D10Rat25-PCR and RT1Ac-flow cytometry, respectively. Data from four sets of experiments were then subjected to the Bland-Altman analysis. As shown in Figure 2(e), all but one data point from the four sets of experiments were located on or within the horizontal lines, which represented the 95% limits of agreement. This finding suggests a good agreement in the performance of these two methods. Specifically, this result demonstrated that the chimerism measured by D10Rat25-PCR can match that measured by flow cytometry and confirmed that the standard curve, although derived from DNA data, is applicable to cell mixtures.

### 3.4. Application of D10Rat25-PCR on VCA Recipients

**Figure 3:**
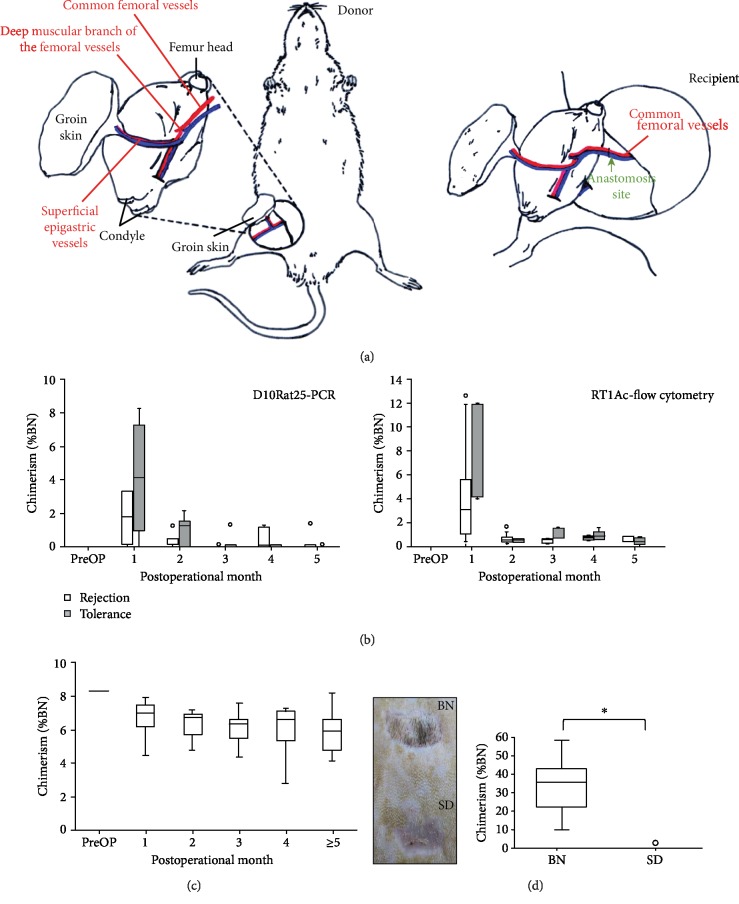
(a) The rat VCA model. The allograft derived from the hindlimb of a BN rat including the intact femur, skin, and muscle was transplanted to the LEW rat. (b) The chimerism of peripheral blood was examined every month following VCA (POM 1-5). The data were grouped according to the final outcome of VCA. The chimerism was elevated at POM 1 then dropped. (c) The VCA skin was biopsied every POM as long as no rejection occurred, and the chimerism was examined by D10Rat25-PCR. The chimerism decreased from 100% to ~75% and maintained at this level till the endpoint. (d) Skin grafts derived from the donor (BN) or the third-party (SD) were transplanted to the recipients with VCA that survived for 120 days. Only the BN skin survived, and the SD skin was rejected (left). The chimerism was examined at 1 month after skin grafting (right).

Next, the method was tested on a VCA model. A BN hindlimb-derived allograft was transplanted to a LEW recipient (Figure 3(a)). The recipients were treated with a short-term immunosuppression regimen composed of one dose of antilymphocyte serum (ALS) at the day before transplantation and 16 mg/kg of cyclosporine from postoperative day (POD) 0 to 10 (*n* = 48 in total). Tolerance to VCA was induced by administration of adipocyte-derived stem cells at POD 1 in addition to immunosuppressants [14]. The recipients were classified based on their VCA outcomes as rejection (*n* = 33) and tolerance (*n* = 15). Chimerism in peripheral blood and the allograft was measured every month after transplantation. We found that peripheral chimerism was elevated from 0 up to ~9% at the first postoperative month and decreased afterwards. No statistical difference was observed between the rejection and tolerance groups (Figure 3(b)). For comparative purposes, we also show the degree of chimerism of peripheral blood measured by flow cytometry with the RT1Ac antibody. Although flow cytometry gave a slightly higher measure of the degree of chimerism, no significant differences were noted between the two methods.

By contrast, chimerism within the allograft dropped at POM 1 from 100% (all BN cells) to 75% in average and then maintained at this level as long as the VCA survived (Figure 3(c)). When the VCA survived for 120 days, secondary skin grafts derived from either BN or SD were transplanted. If donor-specific tolerance developed, the recipient would accept the BN skin and reject the third-party (SD) skin (Figure 3(d), left). Chimerism of the survived BN skin grafts was around 35% in average, whereas the remaining scars following SD skin rejection showed very low chimerism (Figure 3(d), right).

**Figure 4:**
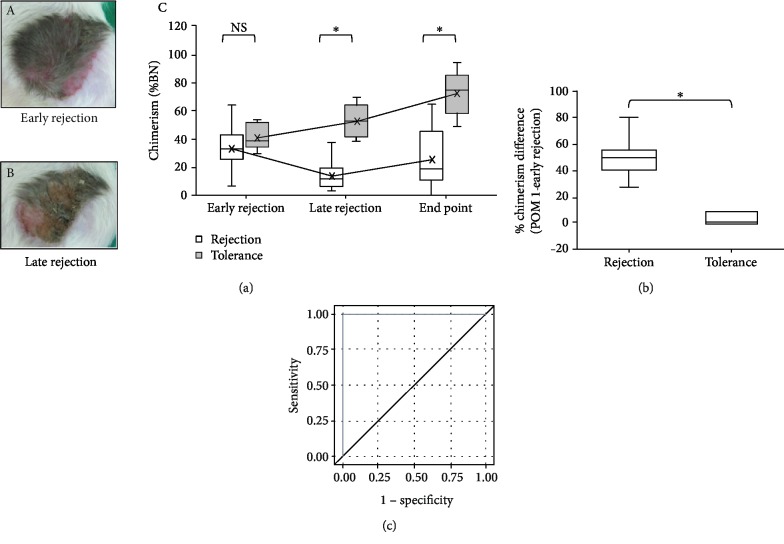
(a) The VCA skin was biopsied at early rejection (A) and late rejection (B), and chimerism was examined with D10Rat25-PCR during rejection and at the endpoint (POM 5) (C). The lines connect the averages of each condition. (b) The differences in chimerism between POM 1 and at early rejection (*Δ*Chimerism_POM1-ER_) were significantly different between the rejection and tolerance groups. (c) The ROC analysis with *Δ*Chimerism_POM1-ER_ showed good sensitivity and specificity to discriminate VCA outcomes. The AUC is 1.

The vast majority of rejection episodes occurred between POM 1 and 2. As shown in Figure 4(a), the average chimerism of the VCA skin decreased to 40% at early rejection, regardless of the final VCA outcome. For those fully rejected VCA, the chimerism kept dropping and ended at about 25% in average. Interestingly, some recipients recovered from rejection, even at the late stage, and the level of chimerism gradually increased to a final average level of 75%. The differences in VCA skin chimerism between the rejection and tolerance groups at late rejection and the endpoint were statistically significant. Further inspection of the data showed that the calculated difference of chimerism between POM 1 and early rejection (*Δ*Chimerism_POM1-ER_) clearly differentiated the rejection group from the tolerance group (Figure 4(b)). For the rejection group, the chimerism dropped dramatically at early rejection, at a magnitude ranging from 26.9% to 81%. However, the tolerance group showed milder changes during early rejection (-0.8% to 8.8%). Receiver operating characteristic curve (ROC) analysis of the *Δ*Chimerism_POM1-ER_ values versus the VCA outcomes showed that the *Δ*Chimerism_POM1-ER_ value has good discriminative power with high specificity and sensitivity. The area under the curve (AUC) equals to 1 (Figure 4(c)). The analysis also estimated the optimum cutoff value to be 17.7%, which has the highest sensitivity and specificity to discriminate the two potential outcomes, i.e., rejection and tolerance.

**Figure 5:**
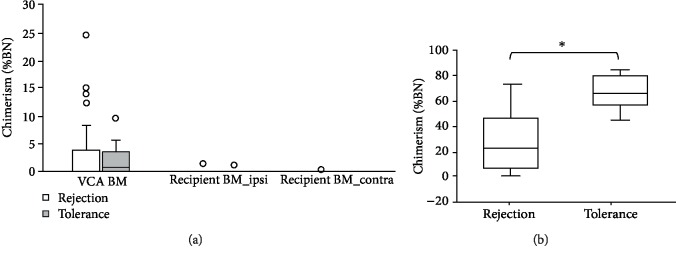
(a) At the experiment endpoint, bone marrow (BM) of the femur in VCA and that of the recipients' own femurs (ipsi: the same side to VCA; contra: the other side to VCA) were isolated to evaluate the chimerism. No differences were observed between the rejection and tolerance groups. (b) The chimerism of the muscle in tolerant VCA was significantly higher than that in the rejected ones at the endpoint.

All recipients were sacrificed after POM 5 (POD 150), and the bone marrow of the recipient femurs and that in the VCA were isolated to examine the chimerism. As shown in Figure 5(a), chimerism of the VCA femur was low and was not statistically different between the two groups at the endpoint. Furthermore, chimerism of both recipient femurs was either undetectable or at very low level.  Figure 5(b) demonstrates that the VCA muscle had the same trend as that of the VCA skin, where the tolerance group had significantly higher level of BN chimerism than the rejection group. For all other internal organs examined, including the heart, liver, and thymus, D10Rat25-PCR showed undetectable chimerism, although the presence of very low chimerism (0.1%) was identified at the spleen of some VCA recipients (data not shown).

## 4. Discussion

Chimerism was first introduced by Owen in 1945 [15]. Anderson et al. noted the importance of a chimeric state to accept dizygotic twin skin graft [16]. These and later observations lead to the belief that the existence of donor cells in the recipient (chimerism) prompts tolerance to donor tissues by the recipient immune system. Chimerism is usually achieved through conditioning of the recipients with irradiation or cell-depleting reagents, followed by infusion of donor-derived cells, especially bone marrow cells. It may be “full” where the recipient hematopoietic system is fully replaced by the donor cells or “mixed” where the donor and recipient bone marrow-derived elements coexist in the recipient [17]. VCA may be performed when the chimerism is established in the recipients at 28 days after irradiation [5] or on the same day when irradiation is performed [18]. Elimination of chimerism with the antibody against donor MHC class I molecule abrogated tolerance, further supporting the critical role of chimerism in tolerance [19].

Although many animal transplantation studies employed flow cytometry and immunohistochemistry for quantitative measurements of chimerism, inconsistency remains as shown at the previous section [3–6]. On the other hand, other methods have been used to measure chimerism, including fluorescent in situ hybridization of sex chromosomes in sex-mismatched transplantations, PCR amplification of variable number of tandem repeats/short tandem repeats (VNTR/STR), single nucleotide polymorphism (allele-specific PCR) [9], and insertion-deletion biallelic polymerism (indels). The detection limit for STR-PCR is generally around 1% and can be as low as 0.024% with indel-PCR [20]. Furthermore, STR-PCR was recommended for chimerism measurement in the guideline published by the United Kingdom National External Quality Assessment Service for Leucocyte Immunophenotyping (UK NEQAS LI) with the advantages of less inter- and intralaboratory variations [21]. In clinical settings, changes of chimerism based on accurate assessment by STR-PCR have been shown with diagnostic potential to track disease relapse following HSCT. Therapeutic intervention can thus be applied early [10].

A few animal SOT studies have employed PCR-based methods to access chimerism. Some took advantage of the sole presence of the SRY gene at the male chromosome to acquire the level of male donor cells in the female recipients [22]. The BN-specific RT1B*β* was also employed for accessing chimerism following kidney transplantation from BN to LEW rats [11]. Both methods measured the level of a donor-specific molecule and provided good sensitivity. However, the quantitative power could be limited because no counterpart molecule in the recipient was measured simultaneously as a reference, which was reflected by our data showing a modest correlation between chimerism and RT1B*β* signal (*R*^2^ = 0.6). In contrast, the D10Rat25-PCR method we described provided superior quantitative power (*R*^2^ > 0.99) by measuring a STR molecule in the donor and the recipient simultaneously.

Chimerism following allotransplantation is mainly due to the migration of donor passenger leukocytes, including dendritic cells and pluripotent stem cells to the recipient lymphoid organs. Here, we demonstrated that peripheral chimerism was transiently elevated to as high as ~9% following VCA regardless of the allograft outcome. Chimerism was mostly undetectable after 3 months, suggesting very few, if any, donor-origin cells were circulating in the recipients. Transient peripheral chimerism was also reported for some nonhuman primate recipients that had developed tolerance to the kidney and lung allograft [23]. After the disappearance of chimerism, graft survival was shown to be maintained with peripheral regulatory mechanisms, such as loss of donor-specific CD8^+^ T cell response and increased proliferation of CD4^+^FoxP3^+^ regulatory T cells, which were specifically induced by donor antigens [24]. It was suggested that the requirement of chimerism to achieve tolerance, being sustained or transient, varied depending on allograft type and recipient species and correlated with the cellular mechanisms that were principally responsible for induction of tolerance to allografts.

Interestingly, the bone marrow in VCA, although originated from the donor, was mostly replaced by the recipient cells (Figure 5(a)). This finding is in line with the earlier report of Muramatsu et al. describing the gradual replacement of allografted bone marrow with the recipient cells in a rat sex-mismatched system. Semiquantitative PCR with Y chromosome-specific primers roughly measured that the residual donor cells were about 10% at 18 weeks after vascularized bone allografting [25]. Although vascularized bone marrow was shown to be critical to induce donor-specific tolerance [26], their roles in maintaining tolerance may not be as significant. In addition to our previous report showing that CD4^+^CD25^+^FoxP3^+^ cells were increased at the recipients that developed tolerance to the VCA [14, 27], we suggest that peripheral mechanisms may be more essential to maintain tolerance at the rat VCA system.

Recipient cells also migrate to the allograft. Starzl and Zinkernagel suggested that the reciprocal clonal exhaustion-deletion accounted for the immunological nonreactivity (tolerance) following such bidirectional cell migration and establishment of chimerism [28, 29]. It was supported by the persistent chimerism observed in patients with stable functioning transplanted liver and kidney, up to 29 years following transplantation [30, 31]. The current D10Rat25-PCR method also provided the advantage to monitor VCA chimerism longitudinally. With this technique, we found that the recipient cells in the allograft such that the BN chimerism dropped from 100% to about 75% on average at POM 1. Chimerism stayed at this level as long as rejection did not happen, suggesting certain recipient cells may play essential roles to maintain VCA survival. Most rejection occurred between POM 1 and 2, and the average VCA chimerism dropped to ~40% during the early stage of rejection. Interestingly, some rejection episodes recovered without any treatment and tolerance later developed. The VCA chimerism during late rejection significantly differentiated the recipients with the final outcome of rejection and tolerance at 10% and 50%, respectively. These data suggested that the onset of rejection was accompanied with more recipient cells localized in VCA, which can be reversed when rejection is ameliorated. The number of recipient cells in VCA increased when rejection continued to progress. Although the VCA-residing recipient cells are likely to be infiltrated by leukocytes, the cell types and characteristics may vary according to the undergoing processes determining allograft fates as a specific time point. Further characterization of the cell identities and requirement of recipient cells in VCA at different stages would provide mechanistic insights and help to devise strategies to promote donor-specific tolerance.

The calculated *Δ*Chimerism_POM1-ER_ of the same recipient showed significantly higher values, ranging from 26.9 to 81% for the rejection group and from -0.8 to 8.8% for the tolerance group. ROC analysis showed high specificity and sensitivity (AUC = 1). The optimum cutoff value with the highest specificity and sensitivity was estimated to be 17.7%. Thus, the predicted VCA outcome is rejection when the *Δ*Chimerism_POM1-ER_ is higher than 17.7%. Additional immunosuppression therapy could be applied in such cases to reverse rejection during early stages. In sum, *Δ*Chimerism_POM1-ER_ may serve as an important guide for immunosuppression therapy as a predictor of VCA outcomes in addition to the clinical symptoms and pathological findings.

Although we have obtained the optimum cutoff value that could potentially be applied to predict VCA outcomes, its predictive power would ideally be further validated by a prospective research design using a different set of VCA recipients and followed by a contingency analysis. Another limitation of our study is the relative high degree of variation in the low percentage of chimerism (ca. 1-3%) that we observed. Application of fluorescence-labelled primers for STR-PCR may enhance the sensitivity, especially for the samples with low chimerism.

## 5. Conclusions

A reliable method to quantify the percentage of BN cells/DNA in BN-LEW chimeras was developed. We successfully characterized the detection limit and ensured the data match those acquired by flow cytometry. The relationship between systematic (in peripheral blood) or local (at specific organ/tissues) chimerism to allograft outcomes was noted. Furthermore, we demonstrated that the magnitude in changes of VCA skin chimerism during early rejection may serve as a predictor of VCA outcome. This technique has application potential in rat VCA and organ transplantation studies. It may complement the cellular characterization data obtained by flow cytometry and the histological information provided by immunohistochemistry, to further depict the requirement, as well as the mechanistic roles of chimerism in detail during the processes of developing transplantation tolerance versus rejection.

## Figures and Tables

**Table 1:** The rat short tandem repeats (STR) with strain differences in size (identified from the Rat Genome Database) [13] screened for the current study.

	D10Rat25	D10Rat46	D10Rat121	D10Rat133
BN/SsNHsd	120	179	183	196
LEW/Pit	142	218	217	162

## Data Availability

The data used and/or analyzed in the current study are available from the corresponding author on reasonable request.

## References

[1] Siemionow M., Nasir S. (2007). Chimerism and bone marrow based therapies in transplantation. *Microsurgery*.

[2] Leventhal J., Abecassis M., Miller J. (2013). Tolerance induction in HLA disparate living donor kidney transplantation by donor stem cell infusion: durable chimerism predicts outcome. *Transplantation*.

[3] Siemionow M., Izycki D., Ozer K., Ozmen S., Klimczak A. (2006). Role of thymus in operational tolerance induction in limb allograft transplant model. *Transplantation*.

[4] Ozer K., Oke R., Gurunluoglu R. (2003). Induction of tolerance to hind limb allografts in rats receiving cyclosporine A and antilymphocyte serum: effect of duration of the treatment. *Transplantation*.

[5] Rahhal D. N., Xu H., Huang W. C. (2009). Dissociation between peripheral blood chimerism and tolerance to hindlimb composite tissue transplants: preferential localization of chimerism in donor bone. *Transplantation*.

[6] Foster R. D., Fan L., Neipp M. (1998). Donor-specific tolerance induction in composite tissue allografts. *American Journal of Surgery*.

[7] Huang W. C., Liao S. K., Wallace C. G., Chang N. J., Lin J. Y., Wei F. C. (2011). Greater efficacy of tolerance induction with cyclosporine versus tacrolimus in composite tissue allotransplants with less myeloablative conditioning. *Plastic and Reconstructive Surgery*.

[8] Streitz M., Miloud T., Kapinsky M. (2013). Standardization of whole blood immune phenotype monitoring for clinical trials: panels and methods from the ONE study. *Transplantation Research*.

[9] Taira C., Matsuda K., Yamaguchi A. (2015). Rapid single nucleotide polymorphism based method for hematopoietic chimerism analysis and monitoring using high-speed droplet allele-specific PCR and allele-specific quantitative PCR. *Clinica Chimica Acta*.

[10] Merzoni J., Ewald G. M., Paz A. A., Daudt L. E., Jobim L. F. J. (2014). Quantification of mixed chimerism allows early therapeutic interventions. *Revista Brasileira de Hematologia e Hemoterapia*.

[11] Noris M., Azzollini N., Mister M. (1999). Peripheral donor leukocytes prolong survival of rat renal allografts. *Kidney International*.

[12] Bland J. M., Altman D. (1986). Statistical methods for assessing agreement between two methods of clinical measurement. *The Lancet*.

[13] Shimoyama M., de Pons J., Hayman G. T. (2015). The Rat Genome Database 2015: genomic, phenotypic and environmental variations and disease. *Nucleic Acids Research*.

[14] Cheng H. Y., Ghetu N., Huang W. C. (2014). Syngeneic adipose-derived stem cells with short-term immunosuppression induce vascularized composite allotransplantation tolerance in rats. *Cytotherapy*.

[15] Owen R. D. (1945). Immunogenetic consequences of vascular anastomoses between bovine twins. *Science*.

[16] Anderson D., Billingham R. E., Lampkin G. H., Medawar P. B. (1951). The use of skin grafting to distinguish between monozygotic and dizygotic twins in cattle. *Heredity*.

[17] Cosimi A. B., Sachs D. H. (2004). Mixed chimerism and transplantation tolerance. *Transplantation*.

[18] Muramatsu K., Kato H., Yoshida Y., Moriya A., Okimoto T., Taguchi T. (2011). Myeloablative irradiation, granulocyte-colony stimulating factor, and FK506 can induce macrochimerism and prolong the survival of experimental extremity allografts. *The Journal of Surgical Research*.

[19] Khan A., Tomita Y., Sykes M. (1996). Thymic dependence of loss of tolerance in mixed allogeneic bone marrow chimeras after depletion of donor antigen. Peripheral mechanisms do not contribute to maintenance of tolerance. *Transplantation*.

[20] Kim S. Y., Jeong M. H., Park N. (2014). Chimerism monitoring after allogeneic hematopoietic stem cell transplantation using quantitative real-time PCR of biallelic insertion/deletion polymorphisms. *The Journal of Molecular Diagnostics*.

[21] Clark J. R., Scott S. D., Jack A. L. (2015). Monitoring of chimerism following allogeneic haematopoietic stem cell transplantation (HSCT): technical recommendations for the use of short tandem repeat (STR) based techniques, on behalf of the United Kingdom National External Quality Assessment Service for Leucocyte Immunophenotyping Chimerism Working Group. *British Journal of Haematology*.

[22] Muramatsu K., Valenzuela R. G., Bishop A. T. (2003). Detection of chimerism following vascularized bone allotransplantation by polymerase chain reaction using a Y-chromosome specific primer. *Journal of Orthopaedic Research*.

[23] Kawai T., Sogawa H., Boskovic S. (2004). CD154 blockade for induction of mixed chimerism and prolonged renal allograft survival in nonhuman primates. *American Journal of Transplantation*.

[24] Hotta K., Aoyama A., Oura T. (2016). Induced regulatory T cells in allograft tolerance via transient mixed chimerism. *JCI Insight*.

[25] Muramatsu K., Kurokawa Y., Kuriyama R., Taguchi T., Bishop A. T. (2005). Gradual graft-cell repopulation with recipient cells following vascularized bone and limb allotransplantation. *Microsurgery*.

[26] Lin C. H., Zhang W., Ng T. W. (2013). Vascularized osteomyocutaneous allografts are permissive to tolerance by induction-based immunomodulatory therapy. *American Journal of Transplantation*.

[27] Lao W. W., Wang Y. L., Ramirez A. E., Cheng H. Y., Wei F. C. (2014). A new rat model for orthotopic abdominal wall allotransplantation. *Plastic and Reconstructive Surgery. Global Open*.

[28] Starzl T. E., Zinkernagel R. M. (1998). Antigen localization and migration in immunity and tolerance. *The New England Journal of Medicine*.

[29] Marcos A., Lakkis F., Starzl T. E. (2006). Tolerance for organ recipients: a clash of paradigms. *Liver Transplantation*.

[30] Starzl T. E., Demetris A. J., Trucco M., Ricordi C., Murase N., Thomson A. W. (1993). The role of cell migration and chimerism in organ transplant acceptance and tolerance induction. *Transplantation Science*.

[31] Starzl T. E., Ramos H., Zeevi A. (1992). Systemic chimerism in human female recipients of male livers. *Lancet*.

